# Proposed Cottage Hospital for Welwyn District

**Published:** 1901-01-05

**Authors:** Henry Burdett


### PROPOSED COTTAGE HOSPITAL FOR WELWYN DISTRICT.

SPEECH BY SIR HENRY BURDETT, K.C.B.

DURING the past ten years, at various periods, the question of a hospital for Welwyn and district has been mooted, but, hitherto, no practical steps have been taken in the matter. There has been no epidemic to stimulate the movement into fresh life, but it is generally felt in the neighbourhood that, owing to the condition of some of the property in which the poor are compelled to live in the existing state of cottage accommodation, some special provision ought to be made for the emergencies of sickness. It has been said that, in the present condition of affairs, if a labourer is stricken with serious illness, it is impossible to hope for a complete restoration to health, and if a hospital were provided such patients could be removed to a place where those opportunities for recovery, which ought to be within the power of all human beings, might be enjoyed. With a view to forwarding this idea, a meeting was held at St. Mary's Hall, Welwyn, on Thursday, December 20th, 1900, when there was a large and influential attendance. The chair was occupied by Col. A. M. Blake, C.B.

The CHAIRMAN, in his introductory remarks, pointed out that they had been called together to consider the advantage of possessing a Cottage Hospital for the three parishes of Welwyn, Codicote, and Digswell. The proposed hospital was for the admission of patients whose means, and the smallness of whose houses did not allow them to derive the benefits which they ought to derive in case of illness, by the use of the best advantages of medical skill at their disposal. He pointed out that some of the cottages were very small and stuffy, and he was sure that if they had a Cottage Hospital the sufferings of these people would be very much mitigated. and that they would be restored to health quicker, and with far greater certainty than they could be under present conditions. Of course, they knew they had two excellent infirmaries: one at Hitchin and one at Hertford. They were very good, but the great drawback was the distance; from Hitchin to Welwyn being nine miles and to Hertford seven miles. He reminded them of the position of a man with a broken leg being taken away to Hitchin or Hertford, and said they could imagine what he would suffer on such a journey. He also pointed to the fact that in the case of patients attending the present hospitals, the distance prevented their friends from visiting them. In a Cottage Hospital at home they would be able to visit them.

Sir HENRY BURDETT, whose speech was received with great interest, said:—Ladies and gentlemen, it gives me very great pleasure to come here this evening, and as I have a duty assigned to me, I propose to commence by discharging that duty—by formally moving "that it is desirable that a cottage hospital should be started in the district."

I am old enough, and have been long enough in hospital work to remember a time when everybody felt that there was a great amount of sympathy for every person who had to go into a hospital, not only because they were sick, but because it was a hospital that they had to go to. That is a condition of affairs which the younger generation of English people cannot understand. In those days—I am speaking of thirty years ago—a hospital was an institution in a crowded community which people often mentioned with bated breath and rather shuddered at, because, you must remember, in those days the mortality in a hospital was serious, if not terrible. When I remind you that for the major operations in a large London hospital the mortality was then from thirty to forty out of every hundred, I give you an idea of the misery, of the dread—the necessary dread with which many people looked upon an entrance to a hospital. But nowadays, thanks to Lord Lister's discovery, the mortality is relatively inappreciable, and the mortality in the hospitals in these cases is something under 3 in 100. That is a marvellous thing, and when you come to remember that all this has been accomplished and carried out within thirty years—within one-third or one-half of the period of our ordinary working life as men and women in this country, you will see what science has done for the whole community through the hospitals. Therefore, I think, on the general question of the hospital, there can be no doubt that the change in public opinion is justified; and I would remind you now that in regard to everyone—I don't care whether he be prince or peasant—the better the education, the fuller the knowledge, the more certain is the conviction in the individual that when sick, or overtaken by accident, or having to undergo an operation, instead of the old adage "There is no place like home," there is no doubt it is true to say in these cases "There is no place like a hospital." And I think myself that this is a very important fact to remember, because the efficiency and equipment of hospitals, and the introduction of that most important factor the trained nurse—the intelligent handmaiden of the doctor—has brought about a revolution with regard to the treatment of sickness and disease which is one of the marvels of the nineteenth century all the world over.

If you will allow me, I will remind you that forty years ago there were no cottage hospitals. A statement like that is useful because it again illustrates the enormous progress of the age in which we live. Forty years ago—that is, to be perfectly accurate, 1859—a country surgeon, in a small village in Surrey, Mr. Albert Napper, F.R.C.S., who was far and away ahead of his time, so far as the intelligence and interest which he applied to the scientific development of his profession was concerned, realised that in order to do his duty to all classes of his patients it was essential that he should have some place where he could treat the severest cases of illness or accident which happened in his immediate neighbourhood, and so he set to work with a great amount of courage to start, and he did succeed in starting, the first cottage hospital at Cranley. He had to bring to bear great ingenuity in converting what had formerly been an old vicarage house—a vicarage house of the type of Oliver Goldsmith's, where a parson was passing rich on £40 a year —and I have often thought that it would be a matter of great interest to preserve that building as one of the national monuments, because I am perfectly certain there are very few things that have originated from the villages which have exercised more power for good upon the establishment and up-keep of the health of the whole community in the rural districts of England.

Whom does a cottage hospital benefit? People say: "Well, it benefits the poor, and as charitable people we will give a sovereign to the cottage hospital because we are showing an interest in the poor." That is not an intelligent way of giving; it is not an intelligent way of supporting an institution, and it won't be an intelligent way of voting for the resolution which I have moved to-night. A cottage hospital will not only benefit the poor; it will benefit all classes—everybody, from the richest to the humblest in the district. You have, nowadays, at the medical schools, higher and higher education, and a higher class of men are sent out to all parts of England from these schools to be doctors in rural places. With that deeper and higher training they have no doubt developed a desire to always continue to be as able medical practitioners as possible, so that they may be able to deal with every kind of case at once and to put it in the best way for making a rapid and satisfactory recovery. If that is so, and it is undoubtedly so, it is perfectly certain that the question as to whether a cottage hospital is needed is not even debateable amongst serious people who take an intelligent interest not merely in the poor, or in the district, but in themselves. Putting it on the lowest basis—putting it on the common ground of providing for one's own, a cottage hospital in every district is an institution which every intelligent person must desire to have and to make as flourishing and prosperous and efficient as possible.

And to show you once more what a cottage hospital may do, I may say that every kind of operation—the most serious, the most difficult—has been undertaken by cottage hospital surgeons in recent years with perfect success, and nurses are attached to these hospitals perfectly competent to nurse the most serious cases into convalescence. And so it has come to pass that now we have some 3,000 or 4,000 members of the medical profession who are living in the rural districts, and are maintaining their efficiency with the aid of the cottage hospital.

Now as to the local side of the question. I put this question to every one of you, and ask you to answer it for yourselves, and if you cannot answer it in the affirmative vote against the resolution. Is the hospital needed? That is the position I want you to take up. You have to look at it from the point of view of your sanitary condition. That is one way to look at it. If your sanitary condition is unsatisfactory; if the dwellings for the poor are unsatisfactory; if you have not got a proper water supply, then I say distinctly that you do want a unit of efficiency which will intelligently illustrate what can be done in the way of providing an efficient small building, with every modern appliance, which shall form an object-lesson which every man and woman may study and understand by learning the initial principles of sanitation, and so come to know where you as a community stand in such matters to-day. I should like to point out further that you would have the advantage of encouraging your medical men in keeping themselves up to the latest developments of their business with the aid of the cottage hospital. And you would have also another advantage. You would have an advantage which is very material to the poor, and also very material to the rich—you would have the nurse. This cottage hospital n its construction should provide accommodation for a district nurse, as well as for the matron, who will have charge of the nursing. Thus in every way you will bring to the homes of the people, who have accommodation, when they are only slightly ill, to remain at home—you will bring to them the means which will be best calculated to expedite their recovery; and in the most serious cases, which will be removed from the cottages to the cottage hospital, you will have the means to benefit and to treat any case of illness, or any case of accident, however severe, with the assured certainty—I am confident from what I know of the medical profession that they will there provide every kind of thing which will do that most important service for the breadwinners—speedily bring them to a state of health when they can return to their daily work, recovered and strengthened by their residence in the hospital wards.

And there will be another and more important service still, and it is this: Every person here in this district will have through the cottage hospital an opportunity of rendering a little personal service to the sick. You may say what you like about having no time, about being full of occupations, about not taking interest, about being too timid to visit, or give any other reason. When you get to the bottom of the inner man or woman, if they lack sympathy in their lives they are only half men and women, and they can never really enjoy and really appreciate the wonders, the beauties and the marvellous opportunities which life offers in a country like this. I myself think this—I have tested it—that in a hospital (I always spend a portion of Christmas Day in some hospital) you see and you realise that members of the staff of the hospitals are so softened and so humanised by attendance on and frequent contact with sickness that they are moved to give up that day themselves to secure that every person in the hospital is made as happy as possible, and helped to realise, as far as possible, all the beauties which we understand to underlie our Christmas festival. For the solitary and depressed person there is no more perfect panacea that I can recommend than to go to the hospital—to go there and see and realise the beauties of the human side of everything in such an institution. Here you can render a little personal service in your own way that may be of service and of profit to yourself. This little hospital—the cottage hospital —would be a centre of unity between all classes of the community, which must tend to build up and to vivify the local life, to the advantage of everybody in this place.

There is one important point to be considered, and that is this: In asking the question, "Is a hospital wanted?" it might also be asked, "Have you got any cases in this district which you could put in a hospital?" "If there have been any cases in the last twelve months which would have recovered more rapidly, would have effected a much more complete cure, and in which the sufferer would have been able to be abroad more speedily if you had had a cottage hospital?" It is a point of great importance. It is one that I raised before I came here in order that I might ascertain the facts. I have on the table a list of the cases which have happened in the last twelve months, and they prove to demonstration that had this cottage hospital been open an immense amount of suffering would have been dispensed with, and further, that you would have had a most useful work for the poor people in the district going on during the whole period. I won't trouble you with the cases, but it is a fact that in a hospital of this size there would have been constant work for the nurse and doctor, and that work would have gone on to the immense benefit and comfort of the individual sufferer and to the advantage of the employer, because the employer would have got the man back to work more speedily and in a more efficient condition.

You have heard from me that a well-administered cottage hospital is very important as an educator on sanitary grounds; that it is a great humanising influence; that it is a centre which unites all classes; that it is an object lesson which has a material effect upon the comfort and the efficiency of everything in the locality in which it is situated, by the educational influence which it must have upon all those who have eyes to see. I am quite confident that when you have taken the trouble to organise this meeting, when you have taken the trouble, being Englishmen and Englishwomen, to have these plans prepared, when you have asked a busy man like me to come out here to try to convey to you—in a very feeble and inefficient way—some of the joys and privileges, and some of the helpfulness which will result to everybody who will only be quickened to a knowledge of the amount of health and comfort which one can get from a right appreciation of other people's sufferings, and a right sense of our duty and the privilege of discharging that duty by giving wisely and supporting such institutions as this cottage hospital, you must admit that by joining heartily in promoting this good work you will derive consolation in the hour of suffering and distress in your own families.

Mr. Montague Price seconded the resolution, which, on being put to the meeting, was carried unanimously.

The Rev. A. C. Headlam then proposed that a committee be appointed to carry out the necessary details, which was done.

